# A Systematic Review of the Impact of Dietary and Lifestyle Factors on Colorectal Cancer Prevention in Gulf Cooperation Council Countries

**DOI:** 10.7759/cureus.69439

**Published:** 2024-09-15

**Authors:** Tasneem Rashed Adam, Basel H Bakhamees, Maryam Abdulla Ali Ahmed Ali, Ahmed M Hamed, Abdulelah Alotaibi, Abdalla Mohamed Hamato, Rawan Taha Zatari, Safaa Abdulmalik Fahad, Ragad Abdulaziz Abdulbari, Haifaa Marzooq Alharbi, Mona Abdelbaky

**Affiliations:** 1 Dental Health, College of Applied Medical Sciences, King Saud University, Riyadh, SAU; 2 Medicine, King Abdulaziz University, Jeddah, SAU; 3 Internal Medicine, Al Jenan Medical Center, A'ali, BHR; 4 Stroke, United Lincolnshire Hospital Trust, Lincolnshire, GBR; 5 Medicine, Taif University, Taif, SAU; 6 Internal Medicine, Shifa Rahima Medical Complex, Ras Tanura, SAU; 7 Family Medicine, Riyadh Second Health Cluster, Riyadh, SAU; 8 Medical Sciences, Ibn Sina National College for Medical Sciences, Jeddah, SAU; 9 Neurology, King Salman Bin Abdulaziz Medical City Madinah Hospital, Al Madinah Al Munawwarah, SAU; 10 Family Medicine, Urwa Health Care Center, Al Madinah Al Munawwarah, SAU; 11 Neonatology, Prince Sultan Military Medical City, Riyadh, SAU

**Keywords:** cancer prevention, colorectal cancer (crc), dietary factors, gcc countries, lifestyle factors, risk factors

## Abstract

Colorectal cancer (CRC) remains a significant health burden in the Gulf Cooperation Council (GCC) countries, necessitating a deeper understanding of modifiable risk factors. Thus, the aim of the study was to evaluate the impact of dietary and lifestyle factors on the prevention of CRC in GCC countries. Studies were identified through electronic searches and reviewed based on relevant keywords. Databases searched included Ovid's MEDLINE, EMBASE, Google Scholar, and Web of Science, covering titles and abstracts published between January 1, 2000 and July 25, 2024. The search strategy encompassed four thematic areas: “colorectal cancer,” “adults above 18,” “risk factors,” and “GCC countries.” The primary focus was on dietary and lifestyle factors. Two reviewers screened titles and abstracts to determine whether the inclusion criteria were met. A total of 1,883 records were identified across these databases. After removing 513 duplicate records, 1,370 records were screened based on titles and abstracts. Of these, 1,284 records were excluded, leaving 86 full-text articles for assessment. Eight studies were ultimately included in the final systematic review, consisting of seven case-control studies and one cross-sectional study. In GCC countries, a diet rich in fruits, vegetables, and fiber has shown protective effects against CRC, while high red meat and refined carbohydrate intake may increase risk. Regular physical activity reduces CRC risk, though the impact of smoking remains inconclusive. Evidence regarding dairy products is contradictory. There is a shortage of high-quality longitudinal studies, highlighting gaps in current research and underscoring the need for larger studies with consistent methodologies.

## Introduction and background

Colorectal cancer (CRC) represents a major global health concern, with approximately 1.9 million new cases and 930,000 deaths reported in 2020 alone [1,2]. The burden varies geographically, with higher incidence rates in developed countries and lower rates in Africa and southern Asia [3]. A recent global study forecasting CRC trends through 2040 has projected a notable increase in cases, particularly in middle- and low-income countries, driven by the rise in sedentary lifestyles and unhealthy dietary patterns. By 2040, the global burden is expected to escalate to 3.2 million new cases and 1.6 million deaths annually [1].

The Gulf Cooperation Council (GCC) countries is a regional political and economic union consisting of six member countries: Bahrain, Kuwait, Oman, Qatar, Saudi Arabia, and the United Arab Emirates (UAE). In GCC countries, the CRC incidence has shown an alarming rise, particularly in Saudi Arabia, where it ranks as the most common cancer in men and the third most common in women [4]. A retrospective analysis of 4,201 CRC cases in the Saudi Cancer Registry from 2001 to 2006 revealed an increasing incidence and a mean diagnosis age of 58 years, with most patients being over 45 [5]. In the UAE, a retrospective descriptive study included 114 patients who underwent surgeries for colorectal carcinoma at Al-Ain and Tawam Hospitals between 1985 and 1998. The study found that the mean annual incidence of CRC in the UAE was 12 patients per year, with a majority being male (67.5%) and an average age of diagnosis at 46.6 years [6]. A recent systematic review reported that Qatar has an annual incidence rate of 7.5 per 100,000 [7]. The most common anatomical site affected is the descending and sigmoid colon [8]. While the overall incidence is lower than in developed countries, Qatar has a higher rate among individuals under 40 years old. 

Numerous studies indicate that diet and lifestyle factors significantly influence the risk of developing CRC. A Western-style diet high in red and processed meats, refined grains, and sugar is associated with an increased risk of CRC [9,10]. Sedentary behavior is also linked to higher colon cancer risk, with a meta-analysis showing a 30% increased risk [11,12], likely due to its contribution to obesity, insulin resistance, and chronic inflammation, all of which are recognized risk factors for CRC. Conversely, protective factors include physical activity, calcium-rich diets, and high consumption of fruits, vegetables, and fiber [9,13]. A case-control study in Egypt found that red meat consumption, preserved foods, and smoking were significant risk factors, while physical activity and a diet rich in fruits, vegetables, and seafood were protective [13]. Additionally, sedentary behavior, particularly prolonged television watching, was linked to elevated colon cancer risk [13]. 

Modifying dietary habits, increasing plant-based food consumption, reducing red meat intake, and maintaining physical activity could substantially reduce CRC incidence and mortality [10,12]. Adopting a balanced diet and maintaining an active lifestyle are critical preventive measures against CRC. Therefore, this review aims to evaluate the impact of dietary and lifestyle factors on the prevention of CRC in GCC countries.

## Review

Method

Search Strategy

We conducted a search to identify published papers on the effects of lifestyle and dietary components on CRC prevention in GCC countries. Databases searched included Ovid's MEDLINE, Embase, Google Scholar, and ISI Web of Science, covering titles and abstracts published between January 1, 2000 and July 25, 2024. The search strategy comprised four thematic areas, incorporating synonyms for “colorectal cancer,” “adults above 18,” “risk factors,” and “GCC countries.” The primary focus was on dietary and lifestyle factors. The search strategy and filters were tailored for each specific database. Additionally, reference lists of the included articles were reviewed to identify further relevant studies.

Study Selection

The initial screening of studies was conducted by reviewing titles and abstracts, followed by a full-text analysis. Duplicates were removed using EndNote 21 (Niles Software, San Jose, CA). The papers were then selected based on the following criteria: peer-reviewed original articles in Arabic or English; case-control, cohort, and cross-sectional studies; research on the incidence, prevalence, or risk of CRC related to lifestyle factors (such as diet, physical activity, smoking, and alcohol consumption); inclusion of individuals diagnosed with CRC as the “case” group and healthy subjects as the “control” group; and studies conducted in GCC nations or containing data specific to GCC populations. Exclusions included editorials, commentaries, case reports, studies lacking sufficient data on lifestyle factors or CRC outcomes, and studies focusing only on the genetic or molecular aspects of colorectal cancer without considering lifestyle factors. Disagreements regarding eligibility criteria were resolved by consulting a third author.

Data Extraction

We extracted data from the research methods and findings of the included studies utilizing an Excel sheet. This data included study characteristics (authors, publication year, sample size/sampling method, and country), participant characteristics (gender and age of participants), dietary factors (specific dietary components and dietary assessment methods), lifestyle factors (physical activity levels, sedentary behavior, measures of obesity, smoking status), CRC outcomes, and key findings.

Risk of Bias Assessment

The risk of bias in the included studies was assessed by two reviewers using the Newcastle-Ottawa Scale (NOS) for quality evaluation of case-control studies [14]. Scores (0 or 1) were given in the Selection and Outcome categories, with a maximum of two stars possible for Comparability. The total maximum score was 10 points. Studies scoring between 10 and 8 points were classified as having a low risk of bias, those scoring between 7 and 5 points as moderate risk, and those with less than 4 points as high risk.

Results and discussion

Literature Search and Study Characteristics

**Figure 1:**
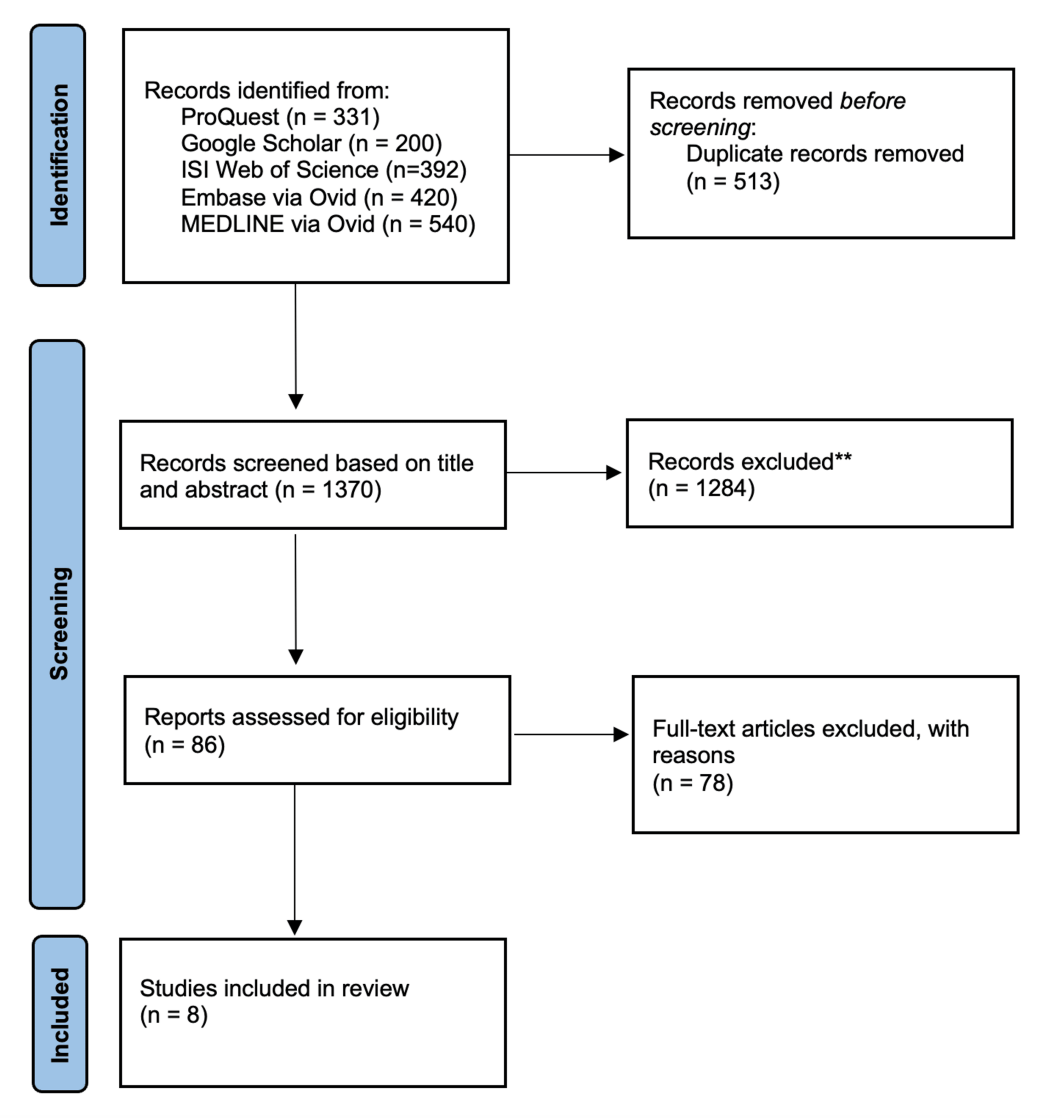
Prisma flow diagram illustrating the study selection process

A total of 1,883 records were identified across these databases: 331 from ProQuest (Ann Arbor, MI), 200 from Google Scholar, 392 from ISI Web of Science, 420 from Embase via Ovid (Elsevier, Netherlands), and 540 from MEDLINE via Ovid (Wolters Kluwer, Netherlands). After removing 1,513 duplicate records, 1,415 records were screened based on titles and abstracts. Following this, 1,329 records were excluded, leaving 86 full-text articles for assessment. Of these eight studies were included in the final systematic review (Figure 1).

Table 1 shows the characteristics of the included studies. Eight studies were conducted in various locations: five in Saudi Arabia, one in Kuwait, one in Oman, and one in Qatar. All the studies employed a case-control design, except for one, which was a cross-sectional retrospective study. Sample sizes varied from 50 to 282 participants, generally spanning adult ages from 18 to 85 years. Both genders were represented in all the studies. Dietary factors such as the consumption of meats, poultry, fish, dairy products, fruits, vegetables, and cereals were analyzed using Food Frequency Questionnaires (FFQs) in most studies, with one study using the Global Physical Activity Questionnaire (WHO) and another using a questionnaire designed by the principal investigator. In terms of lifestyle factors, four studies reported on the impact of physical activity levels in preventing CRC, and three reported on smoking. The findings highlighted several dietary components as either protective or risk factors for CRC. For example, high consumption of lamb and low-fiber foods was associated with an increased risk, while the intake of high-fiber foods, dairy products, and certain vegetables appeared to offer protective benefits. Physical activity levels and obesity measures such as BMI were also examined, with several studies noting a significant association between sedentary lifestyles and higher BMI with increased CRC risk. 

**Table 1:** Characteristics of included studies FFQ - Food Frequency Questionnaire, BMI - Body Mass Index, OR - Odds Ratio, KSA - Kingdom of Saudi Arabia, CI - Confidence Interval, P - p-value.

Study characteristics				Participant characteristics		Dietary factors	Lifestyle factors			Key finding
Study ID	Study location	Study design	Sample size	Age	Gender	Dietary assessment methods	Physical activity levels	Measures of obesity	Smoking status	
Nashar 2008 [15]	King Faisal Specialist Hospital, Riyadh, KSA	Case control study	50 cases and 50 controls	over 30	Both	FFQ	Not mentioned	Not mentioned	Not mentioned	Lamb consumption: OR=13.5 for any consumption, further increasing to OR=3.5 for higher consumption - Chicken with skin and fried eggs: OR=4 and OR=4.93, respectively - Beef consumption: OR=0.18, indicating a protective effect - High-fiber foods: ranging from 0.04 to 0.23, indicating a protective effect against colorectal cancer
Almurshed 2009 [16]	King Faisal Specialist Hospital, Riyadh, KSA	Case control study	50 cases and 50 controls	over 30	Both	FFQ	Exercise/physical activity: (light, moderate, or heavy)	BMI	Not mentioned	No regular exercise vs. Regular exercise: OR = 8.3 (P < 0.05) Light vs. Moderate/Heavy: OR = 8.5 (P < 0.05)
Alamri 2014 [17]	4 different regions (East, Middle, West and South). MOH hospitals, KSA	Case-control study	359 subjects (174 cases, 185 controls)	Age range 20-85 years	Both	FFQ	Inactive, Moderately inactive, Moderately active, Active	BMI	- never smoke - smokers	Bread, Cereal, Rice & Pasta: Significantly less among control than CRC cases group Vegetables and Fruits: significantly higher among control than CRC cases group Meat, Poultry, Fish, and dairy food: No significant association found. Lack of Physical activity 3.21 (CI:1.7 – 5.8) Smoking 1.37 (CI: 0.82 – 2.3) Overweight 0.84 (CI: 0.45-1.5) Obese 1.6 (CI:1.03 – 2.7)
Alkhaldy 2019 [18]	King Abdulaziz University Hospital (KAUH), Riyadh, KSA	cross-sectional retrospective study	61 controls and 40 patients with, colorectal polyps	aged 30e86 years	Both	FFQ	Not mentioned	BMI	Not mentioned	Among the assessed factors, only vegetable intake was significantly higher in patients without colorectal polyps (p = 0.03)
Azzeh 2017 [19]	King Abdullah Medical City Hospital (KAMC), Mecca, KSA	case-control study	137 cases, 164 controls	56.7 ± 8.4 (Control), 56 ± 13.4 (Case)	Both	FFQ	Mentioned as part of the confounding variables but not explicitly detailed	BMI	Mentioned as part of the confounding variables but not clearly detailed	The intake of dairy products, legumes, leafy vegetables, olive oil, black tea, and coffee was found to have a preventive effect against the development of colorectal cancer
Alsheridah 2018 [20]	Kuwait	case-control study	103 CRC cases and 206 matched controls (1:2 ratio)	25-80	Both	global physical activity questionnaire WHO	Physical Activity	BMI	#NAME?	Consuming eggs frequently, consuming red meat frequently, and consuming low fruits and vegetables are risk factors for colorectal cancer. Lack of physical activity, obesity, and a BMI of 30 or higher are also significant risk factors. Preventive factors include higher fruit and vegetable intake and adequate physical activity.
Mafiana 2018 [21]	Sultan Qaboos University Hospital (SQUH), Muscat, Oman	Case-control study.	109 cases and 179 controls	18 years and above	Both	FFQ	Sedentary or Active	BMI	Never or Former/Current	Overweight individuals have a higher risk of developing CRC compared to those with normal weight. Consumption of vegetables and fruits is lower in CRC cases, while proteins, fat, and carbohydrates are higher. No significant association was found between smoking or alcohol consumption.
Bener 2016 [22]	Al-Amal Cancer hospital and primary health care centers in Qatar	Case-control study.	Case N=146 Control N=282	40-60	Both	A questionnaire was designed by the principal investigator	Not mentioned	BMI	smokers - non smokers	Bakery products: 2.52 (2.30 - 2.88) <0.0001 BMI: 2.30 (1.38 - 3.83) <0.0001 Smoking: 2.12 (1.12 - 3.85) 0.017 Soft drinks: 1.62 (1.19 - 2.17) 0.020 Other factors analysed in the study were not found to be significant.

Quality of the Included Study

Overall, the included studies demonstrated good quality as measured by the NOS Quality Assessment Scale (Table 2). Two studies scored 8 points on the scale, five studies scored 6 points, and one study scored 5 points. All the included studies received no points for the “Ascertainment of Exposure” criterion because they relied on self-reported questionnaires to measure exposure.

**Table 2:** Newcastle-Ottawa Scale (NOS) for assessing the quality of case-control studies A single star (★) indicates a study meets the criteria for a specific domain. Two stars (★★) are awarded only for the “Comparability of cases and controls” and “Ascertainment of exposure” criterion.

	Selection				Comparability	Exposure				
Study	Is the case definition adequate?	Representativeness of the cases	Selection of controls	Definition of controls	Comparability of cases and controls	Ascertainment of exposure	Ascertainment for cases and controls	Non-response rate	Total
Nashar et al. 2008 [15]	★	★	0	★	★★	0	★	0	6/10
Almurshed et al. 2009 [16]	★	★	0	★	★★	0	★	0	6/10
Alamri et al. 2014 [17]	★	★	0	★	★★	0	★	0	6/10
Alkhaldy 2019 [18]	★	★	0	★	★	0	★	0	5/10
Azzeh et al. 2017 [19]	★	★	0	★	★★	0	★	0	6/10
Alsheridah et al. 2018 [20]	★	★	0	★	★★	0	★	0	6/10
Mafiana et al. 2018 [21]	★	★	★	★	★★	0	★	★	8/10
Bener et al. 2016 [22]	★	★	★	★	★★	0	★	★	8/10

Studies on diet and colon cancer

Fruits and Vegetables

**Figure 2:**
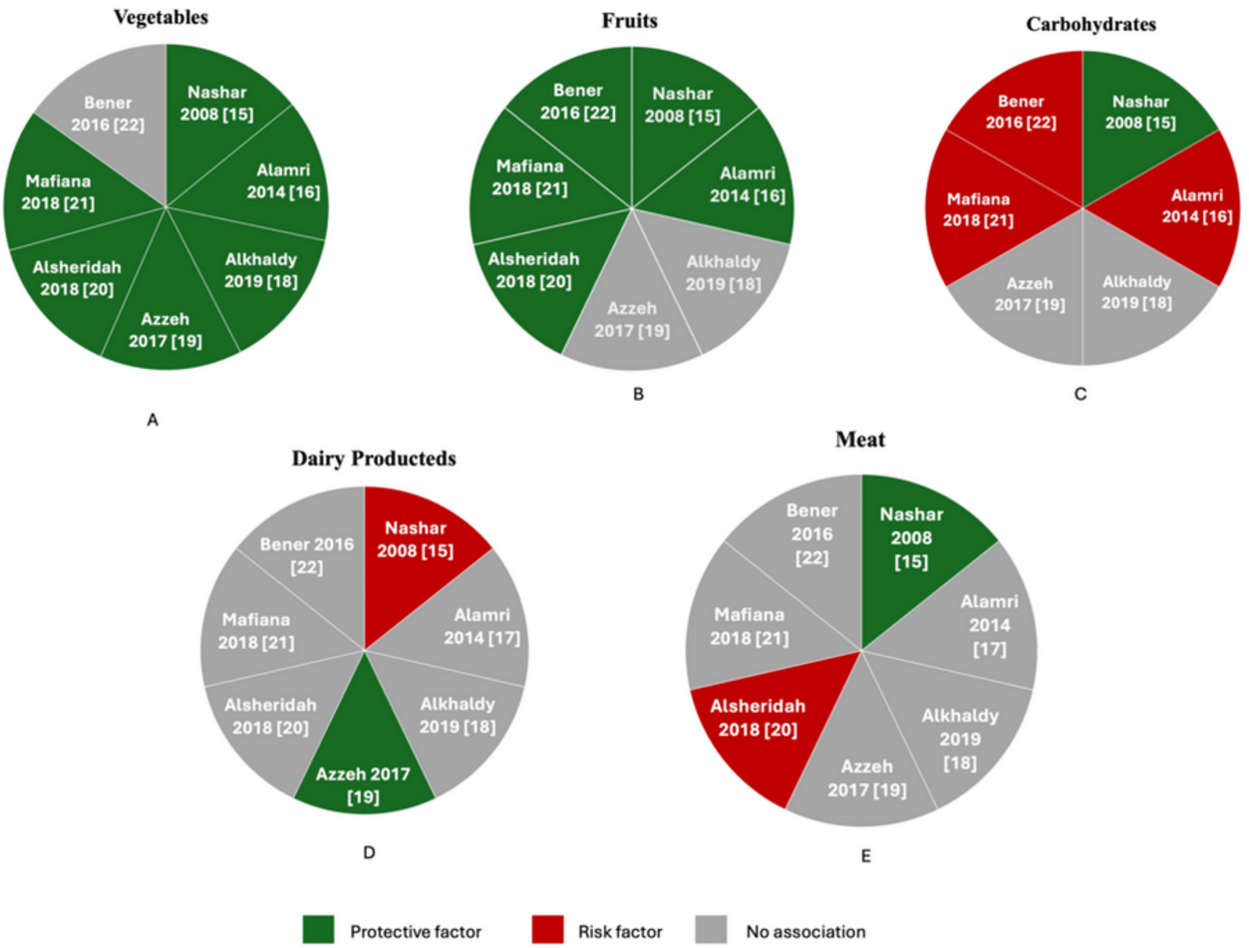
(A-E) Pie charts illustrating the dietary factors associated with CRC prevention as reported in the included studies

A substantial body of literature has demonstrated that a diet high in vegetables and fruits has a protective effect against CRC (Figures 2A, 2B) [15,17-21]. Foods rich in fiber may help reduce this risk by shortening the duration of exposure between fecal carcinogens and the colon mucosa and by decreasing bowel transit time. Alkhaldy et al. found that patients without colorectal polyps adhered more closely to dietary guidelines for vegetable intake compared to those with polyps [18]. Another study conducted in Kuwait reported a significant association between low consumption of fruits and vegetables (never or less than once per day) and increased CRC risk, consistent with findings from other studies in Middle Eastern countries [20]. A case-control study conducted in Jordan reported that a diet low in fruits and vegetables, and high in red meat and saturated fats, was associated with CRC among Jordanian subjects [23]. Furthermore, a meta-analysis emphasized that even modest increases in fruit and vegetable consumption can lower CRC risk, particularly when starting from low baseline levels [24]. However, not all research supports this protective effect. Some studies have found no significant association between fruit and vegetable intake and CRC risk. For example, Bener et al. found no correlation between vegetable intake and CRC [22]. Interestingly, most cases in their study reported a high and regular intake of fruits, vegetables, and green salads. Instead, the study highlighted genetics as a major risk factor, with over 40% of the cases having a family history of the disease compared to the control group [22].

Meat

Nashar et al. discovered an association between lamb consumption and CRC with an OR of 13.5 for any level of intake, which increased to an OR of 3.5 with higher consumption (Figure 2C) [15]. The research was conducted among Saudis, where lamb is commonly consumed with rice and a sweet beverage, often with minimal vegetable intake. This observation suggests that other potential confounding factors should be considered. Interestingly, the authors also found that consuming beef had a protective effect, indicating that meat itself may not inherently increase cancer risk. Instead, the dietary and lifestyle patterns linked with high red meat intake might contribute to a higher risk of CRC. For example, individuals with diets high in red meat and fat may not be following a healthy diet or lifestyle overall. Indeed, Alsheridahet al. reported that a higher risk of CRC was linked to obesity, consuming an excessive amount of red meat, and eating fewer fruits and vegetables [20]. However, most of the included studies did not find a relationship between meat consumption and CRC, which aligns with other published papers. A meta-analysis of prospective studies and a large cohort study conducted among health professionals in the USA found that while processed red meat is positively associated with CRC risk, particularly distal colon cancer, unprocessed red meat shows a weaker and non-significant association with CRC [25,26].

Carbohydrates and Dairy Products

Among the six studies, Nashar et al. reported a potential protective effect of carbohydrates on CRC (Figures 2D, 2E). However, it is important to note that the carbohydrates examined were whole wheat bread and bran, which are rich in fiber. The protective effect observed might therefore be attributed not to carbohydrates in general, but specifically to the high fiber content of these foods [15]. On the other hand, three studies [17,21,22] identified a potential risk associated with carbohydrate consumption, particularly when the intake involved refined carbohydrates and sugary foods. These findings suggest that not all carbohydrates have the same impact on CRC risk, with refined carbohydrates potentially contributing to increased risk. Meanwhile, the studies by Azzah et al. and Alkhaldy et al. found no significant association between carbohydrate intake and CRC [18,19].

As for dairy products, various research papers have examined whether they have a protective effect against CRC or if they contribute to its risk. Nashar et al. found that certain dairy products might increase CRC risk, potentially due to components like high-fat content. For instance, whole milk consumption was associated with a significantly higher CRC risk (OR=9.88, p<0.04; OR=10.5, p<0.03). While moderate Laban intake did not show a significant increase in risk, consuming it four or more times per week raised the CRC risk 18-fold (OR=18.6, p<0.01). Similarly, frequent labneh consumption increased CRC risk 24-fold compared to intake of 2-3 times per week (OR=24, p<0.02) [15]. In contrast, Azzah et al. reported a potential protective effect of dairy products against CRC, likely due to the presence of beneficial nutrients such as calcium and vitamin D. The study suggests that higher dairy intake may be linked to a reduced risk of developing CRC [19]. This finding aligns with recent evidence, which also indicates that increased consumption of total dairy products and milk is associated with a lower CRC risk [27-29]. However, other studies, including those by Bener et al., Mafiana et al., Alamri et al., Alsheridah et al., and Alkhaldy et al., found no significant association between dairy intake and CRC risk, which contrasts with the emerging consensus on the protective effects of dairy. This inconsistency might be because all the studies were case-control studies, which often have limitations in establishing strong relationships due to their design [27].

Studies on lifestyle and colon cancer

Physical Activity, BMI, and Smoking

**Figure 3:**
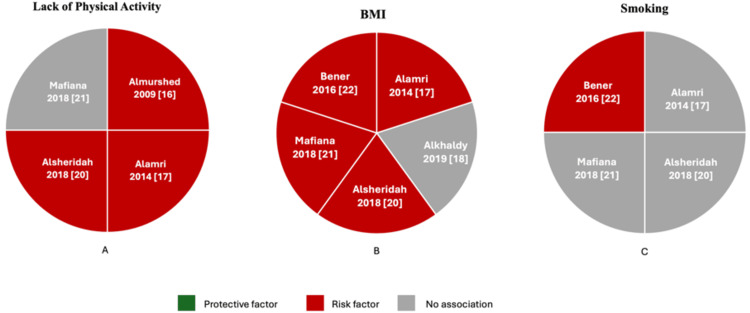
(A-C) Pie charts illustrating the lifestyle factors associated with CRC prevention as reported in the included studies

Only four studies have investigated the impact of physical activity on CRC (Figure 3A). Among these, three reported a significant association between lack of physical activity and an increased risk of CRC. Almurshed found that 90% of cancer cases occurred in individuals who did not engage in regular exercise [16]. Previous research has consistently demonstrated that physical activity reduces the risk of colon cancer and is effective in preventing other cancers. A meta-analysis by Wolin et al. found an association between physical activity and a reduced risk of colon cancer, showing that engaging in physical activity lowers the risk by around 24% overall [12]. Therefore, a sedentary lifestyle is strongly associated with an increased risk of colon cancer. However, Mafiana et al. did not find a significant link between physical inactivity and CRC risk. Nevertheless, their study showed that being overweight, compared to having a normal weight, was associated with more than a threefold increase in the risk of CRC [21]. Indeed, all the included studies that examined the impact of BMI on CRC reported a strong relationship, except for one study by Alkhaldy, which did not find an association across all BMI groups-likely due to the small sample size [18]. A recent review of 38 longitudinal studies found a significant link between overweight, obesity, and CRC, showing an 8% increase in CRC risk for every 5 kg/m² rise in BMI (Figure 3B) [30]. 

Smoking, on the other hand, appears to have no relationship among Gulf citizens with CRC (Figure 3C). Out of four studies, only one found a significant association between smoking and CRC. A prospective cohort study found that smoking cigarettes is associated with an increased risk of developing tumors that could lead to CRC [31]. Moreover, a 2020 review of the Japanese population found that smoking increases the risk of colon cancer in men and distal colon cancer in women [32]. Additionally, another study aimed at assessing the dose-response relationship concluded that cigarette smoking increases the risk of CRC in a dose-dependent manner, with risk rising with both the intensity and duration of smoking. Importantly, quitting smoking was shown to reduce the risk of CRC [33]. 

Limitations

The results of this study include some limitations that should be considered when interpreting the findings. First, most of the included studies were observational, specifically case-control designs, which are prone to recall bias. Second, there was considerable variability in the assessment methods for diet and physical activity across studies, which may have contributed to inconsistencies in the findings. Additionally, most studies included in this review were conducted in KSA, with limited generalizability to other countries within the GCC region. Furthermore, the relatively small sample sizes in several studies may have reduced the statistical power to detect significant associations, particularly for subgroups based on gender, ethnicity, genetics, or age. Finally, most studies that reported on smoking factors did not include information on the duration and intensity of smoking. Additionally, they categorized subjects simply as smokers or non-smokers, without considering former smokers who might have quit only one or two months prior-which could have contributed to the lack of association found between smoking and CRC.

## Conclusions

To the best of our knowledge, this is the first systematic review to specifically investigate the impact of dietary and lifestyle factors on CRC prevention in GCC countries. This review synthesizes existing research on how these factors influence CRC risk within the GCC. While extensive research has concentrated on curative treatments and surgical interventions, prevention is ultimately more effective than treatment. Despite this, prioritizing prevention remains critical to reducing the burden on both the population and healthcare systems. The review emphasizes significant associations between diet, lifestyle, and CRC risk, highlighting the crucial role of modifiable factors in cancer prevention.

In GCC countries, dietary and lifestyle factors significantly influence CRC prevention. A diet rich in fruits, vegetables, and fiber has demonstrated a protective effect against CRC, whereas high consumption of red meat, refined carbohydrates, and certain dairy products may elevate risk. Regular physical activity is consistently associated with reduced CRC risk, highlighting the dangers of a sedentary lifestyle. Although smoking's impact on CRC remains unclear among Gulf populations, it has been linked to increased CRC risk in other regions. The evidence underscores the importance of promoting healthy dietary patterns and active lifestyles to reduce CRC incidence in the region. Research gaps highlight the need for larger, diverse studies and consistent methodologies in assessing dietary and lifestyle factors to tailor effective prevention strategies.
